# Change of termite hindgut metabolome and bacteria after captivity indicates the hindgut microbiota provides nutritional factors to the host

**DOI:** 10.3389/fbioe.2023.1228918

**Published:** 2024-01-15

**Authors:** Most Shormi Alom, Yijing Cen, Rui Tang, Dasong Chen, Hongliang Dou, Zhenzuan Mo, He Du

**Affiliations:** ^1^ Guangdong Key Laboratory of Integrated Pest Management in Agriculture, Guangdong Public Laboratory of Wild Animal Conservation and Utilization, Institute of Zoology, Guangdong Academy of Sciences, Guangzhou, China; ^2^ College of Plant Protection, South China Agricultural University, Guangzhou, China; ^3^ Guangdong Provincial Key Laboratory of Silviculture, Protection and Utilization, Guangdong Academy of Forestry, Guangzhou, China

**Keywords:** symbiosis, nitrogen economy, metabolite, rearing condition, uricolytic bacteria

## Abstract

The gut-dwelling microbiota is an indispensable part of termites. It is influenced by a series of factors, such as diet and captivity. The objectives of this study were to study the metabolic functions of hindgut microbiota and to investigate the influence of captivity on the hindgut microbiota. The dampwood termite *Hodotermopsis sjostedti* was reared in the laboratory for 6 months. We conducted the metabolome analysis of the fat body from the freshly-collected workers (FBF), the hindgut fluid of the freshly-collected workers (HFF), and the hindgut fluid of laboratory-maintained workers. In addition, the 16S rRNA genes from the hindgut bacteria in the freshly-collected and laboratory-maintained workers were sequenced. According to our results, the concentrations of metabolites associated with amino acid biosynthesis, vitamin biosynthesis, fatty acid biosynthesis, and cofactor biosynthesis were higher in HFF compared with those in FBF, suggesting that the hindgut microbiota provides nutritional factors to the host. However, after captivity, the concentrations of metabolites in the hindgut associated with amino acid biosynthesis, nucleotide sugar metabolism, vitamin biosynthesis, and carbon metabolism decreased, while those associated with the steroid hormone biosynthesis and ovarian steroidogenesis increased. Meanwhile, the 16S amplicon study revealed that the abundance of certain bacteria changed after captivity, such as uncultured Termite Group 1 bacterium, *Candidatus* Symbiothrix dinenymphae, and unclassified *Desulfovibrio*. Our findings show that captivity influences the hindgut microbiota and shed light on the metabolic potential of the hindgut microbiota.

## Introduction

Termites’ capability of wood decomposition relies on the mutual symbiosis with their diverse hindgut microbiota. The hindguts of termites are enlarged to harbor an extensive array of microbiota. The protist-dependent termites, which include all termites except those from the family Termitidae, have both bacteria and protists in their guts, while only bacteria exist in Termitidae (Carrijo et al., 2023). The gut microbiota is an indispensable part of termites, and plays a significant role in lignocellulose digestion. The protist-dependent termite *Coptotermes formosanus*, harbors five species of protists in the hindgut, each of which can produce specific enzymes for lignocellulose digestion (Nishimura et al., 2020). Similarly, the bacteria in the hindguts of Termitidae are also involved in lignocellulose digestion (Warnecke, et al., 2007; Liu, et al., 2019; Calusinska et al., 2020). Besides lignocellulose digestion, the hindgut microbiota provides nutritional factors for the host, such as amino acid and vitamin, making the gut microbiota an indispensable part of termites. For example, endosymbiont bacteria from the protists *Eucomonympha* and *Pseudotrichonympha grassii* conduct acetogenesis and nitrogen fixation (Hongoh et al., 2008; Ohkuma et al., 2015). The genomes of these bacteria encode the genes for amino acids and cofactors synthesis, indicating that they may provide these nutritional factors for the hosts.

Wood is known to be short of nitrogen, the component of protein and nucleic acid, so fixation of atmospheric nitrogen by utilizing the gut bacteria is crucial for termites in order to maintain the nitrogen economy (Breznak et al., 1973). In addition, there is also cannibalism inside the colony, in which termites recycle the nitrogen through feeding on the dead nestmates. Finally, termites also take advantage of the uric acid, the metabolic waste of purine, through the bacteria in the guts (Potrikus and Breznak, 1980a). The gut bacteria degrade the uric acid and convert it to the nutrition that can be utilized by termites (Potrikus and Breznak, 1981). The uric acid concentration in the fat body is maintained at a relatively stable level in the field colony. However, when termites are maintained in the laboratory, the uric acid concentration gradually increases (Potrikus and Breznak, 1980b; Lovelock et al., 1985). This means that captivity of termites may influence the uric acid-utilizing bacteria, and thus influences the recycle of uric acid.

Termite colonies are complex adaptive systems influenced by both internal and external factors (Bonabeau, 1998), with division of labor as a distinguished characteristic. There are three general castes inside the colony, i.e., workers, soldiers, and reproductives. The caste differentiation is driven by hormones within the individuals, such as juvenile hormone. Termites use semiochemicals to maintain the caste composition among the colony members (Matsuura et al., 2010). For example, the queen pheromone can control worker behaviors and inhibit workers’ differentiation into the secondary reproductives (Funaro et al., 2018). As a result, the removal of the queen from the colony can trigger workers’ butting behavior (Korb et al., 2009), and later the workers gradually differentiate into the secondary queen or king (Shimoji et al., 2017). Although caste differentiation in termites is controlled by the internal changes of hormones, there is evidence that the hindgut microbiota is involved in the process (Sapkota et al., 2021; Scharf and Peterson, 2021).

The rearing condition, such as food, can exert an impact on the bacteria composition of the *C. formosanus* (Husseneder et al., 2009). The same phenomenon was also observed in Termitidae (Mikaelyan et al., 2015; Wang et al., 2016). In addition, the protists in the hindguts are also affected by the diet composition (Tanaka et al., 2006). It is well known that captivity influences the composition of gut microbiota. For example, the gut microbiota is different between the laboratory-reared German cockroaches and the field-collected ones (Kakumanu et al., 2018). Change of bacteria in the ceca has also been recorded in the wild and captive birds (Wienemann et al., 2011). Captivity can influence the mammalian gut microbiota, too (McKenzie et al., 2017). In addition, captivity of the *Cortaritermes intermedius* in the laboratory significantly changes the species richness and diversity of the gut bacteria (Calusinska et al., 2020). Therefore, the change of gut microbiota occurs with the change in environment when termites are maintained in the laboratory.

To get a more comprehensive understanding of the metabolic functions of termite hindgut microbiota and the influence of laboratory captivity on the hindgut microbiota, we performed the metabolome analysis of the fat body and hindgut fluid in the dampwood termites *Hodotermopsis sjostedti*. The 16S rRNA gene sequencing was also performed for the hindgut bacteria from the freshly-collected termites and the termites maintained in the laboratory. Our findings shed light on the metabolic potential of the hindgut microbiota.

## Materials and methods

### Bioassay

Six colonies of the dampwood termite *H. sjostedti* were collected in Mangshan National Forest Park, Hunan, China. A section of the wood log which was infested by *H. sjostedi* was collected. The logs were kept in a plastic container (51 × 35 × 30 cm) at 25°C in the laboratory, and water was sprayed onto the log regularly to maintain the moisture of the wood.

Wooden slices (15 × 10 × 0.5 cm) and sticks (15 × 1.5 × 0.5 cm) of pine wood were soaked in water overnight. Two wooden sticks were placed at the bottom of a plastic box (19 × 13 × 7 cm), about 6 cm apart from each other. Then, a wooden slice was placed on the wooden sticks. Afterwards, two wooden sticks were placed on the wooden slice, with the same spacing of 6 cm. Using this method, three pieces of wooden slices were piled together. Several holes were punched on the lid of the plastic box for aeration. A number of 200 workers (6th instar or 7th instar) and five soldiers were selected randomly and kept in the box. All the boxes were maintained at 25°C. The termites were reared for 6 months before sample collection. After 6 months of captivity, termites showed an obvious sign of uric acid accumulation (Xing, et al., 2014; Arango et al., 2017). In summary, there were two groups of termites: the freshly-collected termites (F) and the laboratory-maintained termites (L).

### Sample collection

Termites were anesthetized on ice. An individual termite was sterilized in 70% alcohol and then washed in sterilized water. Its gut was pulled out with one tweezer holding the head and another pulling the gut out from the end of the abdomen. Then, the termite was dissected using tweezers. The fat body was harvested using tweezers and stored at −80°C individually. Fat body from the freshly-collected workers (FBF) was collected. Afterwards, the hindgut paunch was broken with tweezers. The gut fluid from a hindgut was sucked with a pipette, and immediately immersed into liquid nitrogen and stored at −80°C individually. Two groups of hindgut fluid were used in this experiment: one group was the hindgut fluid from the freshly-collected workers (HFF) and the other was that from the laboratory-maintained workers (HFL). There were six colonies for each group, with one colony as a replicate (Jiang et al., 2022).

The hindguts of workers were dissected. Three hindguts were pooled together in the experiment, and their DNA was extracted using the Omega E.Z.N.A.^®^ Stool DNA Kit (D4015-01) according to the manufacturer’s protocol. Two groups of DNA were extracted, namely, the DNA from the hindguts of the F group and the DNA from the L group. There were five colonies for each group, with one colony as a replicate.

### Metabolome analysis

The fat body and hindgut fluid were pooled together, respectively, and freeze-dried using a lyophilizer. The weights of the freeze-dried samples were weighed and methanol-acetonitrile-water (2:2:1, v/v/v; internal standard = 20 mg/L) was added in proportion to the weight in order to equalize concentrations of the samples. 2-chloro-L-phenylalanine was used as the internal standard. The solutions were homogenized by a grinder with metal beads, followed by treatment in ultrasound bath for 10 min and incubation for 1 h at −20°C. The samples were then centrifuged at 12,000 rpm for 15 min at 4°C. The resulting supernatants were dried in a centrifugal vacuum concentrator, and then the dried samples were dissolved in acetonitrile-water (1:1, v/v), vortexed for 30 min, and treated with ultrasonic bath for 10 min. Then, the supernatants were collected by centrifugation. The quality control (QC) sample was prepared by pooling 10 μL of the supernatants from all the samples. The QC sample was analyzed to provide robust quality assurance. The supernatants were analyzed with Acquity I-Class PLUS coupled with Xevo G2-XS QTof. The injection volume was 1 μL. Separation was performed on an Acquity UPLC HSS T3 column (1.8 μm, 2.1*100 mm; Waters) using water (0.1% formic acid; mobile phase A)-acetonitrile (0.1% formic acid; mobile phase B) at a flow rate of 400 μL/min. The elution gradient program was as follows: 0 min, 98% A, 2% B; 0.25 min, 98% A, 2% B; 10.0 min, 2% A, 98% B; 13.0 min, 2% A, 98% B; 13.1 min, 98% A, 2% B; 15.0 min, 98% A, 2% B. Under the supervision of the acquisition software, the Waters Xevo G2-XS QTOF high resolution mass spectrometer collected primary and secondary mass spectrometry data in MSe mode (MassLynx V4.2, Waters). Dual-channel data acquisition can be performed on both low collision energy and high collision energy at the same time throughout each data acquisition cycle. For a mass spectrum, the low collision energy was 2 V, the high collision energy range was 10∼40 V, and the scanning frequency was 0.2 s. The ESI ion source’s parameters were as follows: capillary voltage: 2000 V (positive ion mode) or 1500 V (negative ion mode); cone voltage: 30 V; ion source temperature: 150°C; desolvent gas temperature 500°C; backflush gas flow rate: 50 L/h; desolventizing gas flow rate: 800 L/h.

### 16S amplicon sequencing

The library construction and sequencing were as follows. To identify the amplicons from different samples, the barcodes were added to the primers 27F and 1492R (27F: 5′-AGRGTTTGATYNTGGCTCAG-3′; 1492R: 5′-TASGGHTACCTTGTTASGACTT-3′). The primers were used to amplify the full length 16S rRNA genes. The thermal cycling conditions were 95°C for 2 min, followed by 25 cycles of 98°C for 10 s, 55°C for 30 s, and 72°C for 1 min 30 s, and then followed by a final extension of 72°C for 2 min. The amplification products were purified, quantified and homogenized to generate the SMRTbell libraries. After library quality control, the qualified libraries were sequenced on PacBio Sequel Ⅱ platform. The bam format files generated by the Pacbio Sequel Ⅱ were converted into CCS (Circular Consensus Sequencing) files by smrtlink. CCS produces a highly accurate consensus sequence from multiple reading of a single SMRTbell molecule (Wenger et al., 2019). The sequences from each sample were identified by the barcode sequences and converted into the fastq sequences.

### Data analysis

The original data collected by MassLynx were imported into Progenesis QI for retention time calibration, peak identification, peak extraction and peak integration, and peak alignment, etc. The metabolites were identified based on accurate mass, retention time, and tandem MS data using the METLIN database and a self-established database (Biomarker Technologies Corporation, Beijing). For theoretical fragment identification, the parent ion mass deviation was set within 100 ppm and fragment ions mass deviation was within 50 ppm. The quantification of the metabolites was processed using MetaboAnalystR (Chong and Xia, 2018). The metabolites were then annotated using the Kyoto Encyclopedia of Genes and Genomes database (KEGG) (Kanehisa and Goto, 2000), Human Metabolome Database (HMDB) (Wishart et al., 2018), and Lipid Metabolites and Pathways Strategy (LIPID MAPS) (Fahy et al., 2007). Afterwards, the fold change of each metabolite between two groups was calculated.

To respectively explore the metabolic differences between FBF and HFF, and those between HFF and HFL, the orthogonal partial least squares discriminant analysis (OPLS-DA) was performed (Thévenot et al., 2015). The OPLS-DA is a supervised multivariate data analysis method. The orthogonal variates unrelated to categorical variates were removed from the data set. To validate the robustness of the OPLS-DA model, a random permutation test was performed. To screen the differentially abundant metabolites, a combination of fold change, *P*-value in the student’s t test, and the variable importance in projection (VIP) value of the OPLS-DA was used. The criteria were FC > 1, *P* < 0.05, and VIP > 1. The differentially abundant metabolites were summarized in a volcano plot. The differentially abundant metabolites were analyzed with hierarchical clustering analysis. To identify the function of the differentially abundant metabolites, the KEGG pathway enrichment analysis was performed (Yu et al., 2012). Fisher’s exact test was used to perform the KEGG pathway enrichment analysis.

The original bam format data were converted into CCS files by smrtlink through CCS identification, CCS filtration, and Chimera removal as follows. Raw CCS sequences were generated by identification barcode sequences using lima v1.7.0. Then, cutadapt 1.9.1 was used to remove the primer sequences and to conduct length filtration to obtain the clean CCS sequences (Martin, 2011). UCHIME v4.2 was used to remove the chimera to generate the effective CCS sequences (Edgar et al., 2011). The sequences for different samples were identified by the barcode sequences and transformed into fastq format data. The quality of the sequences was evaluated in terms of the number of the sequences in each processing stage.

To explore the taxonomic composition of the hindgut bacteria, the 16S amplicon sequences were first categorized into operational taxonomic unit (OTU) based on sequence similarity. The OTUs were obtained based on 97% similarity with Usearch (Edgar, 2013). One OTU corresponded to one representative sequence. Venn diagram was used to display the relationship among replicates in a group (Chen and Boutros, 2011). To identify the taxonomy of each OTU, the taxonomic annotation and analysis were performed. Naive Bayes classifier was used to annotate the characteristic sequence using the SILVA database (release 132) (Quast et al., 2012). Then, the composition for each sample was calculated at the phylum, class, order, family, genus, and species level. The abundance at each taxonomic level was calculated using QIIME (Bolyen et al., 2019). The bacterial community composition histogram was drawn using R.

Alpha diversity reflects the richness and diversity of each species in a given community. The evaluation indices include Chao1, ACE, Shannon, Simpson, and PD whole tree. Chao1 and ACE were used to evaluate the species richness. Shannon index, Simpson index, and PD whole tree were used to evaluate the species richness and evenness. The alpha diversity indices were calculated using QIIME2 (version 2020.6). The differences of the alpha diversity indices between F and L group were tested by Mann-Whitney U test. To evaluate the similarity of species diversity between the treatments, the beta diversity analysis was performed using Bray Curtis distance metric. Beta diversity is used to compare the similarity of the species diversity between samples. Principal coordinates analysis (PCoA) was used to visualize the beta diversity analysis using R (Dubois et al., 2010).

For the purpose of analyzing the hindgut bacteria difference between the F group and the L group, Metastats analysis (White et al., 2009) and LEfSe (Line Discriminant Analysis (LDA) Effect Size) (Segata et al., 2011) were performed. The FDR adjusted *P*-value (*Q* value) was used to determine significance in the Metastats analysis (*Q* < 0.05). LEfSe is an analysis method to detect and interpret the high-dimensional biomarker, such as genes, pathways, and clades. It is used to detect the features (or biomarkers) which are most likely to interpret the between-group differences, and the extent to which these biomarkers contribute to the difference. The data were subjected to LEfSe analysis to find out the discriminative features in the samples. To study the correlation between different bacteria, Spearman rank correlation was performed. The correlation results whose ρ > 0.1 and *P* < 0.05 were used to construct the correlation network (Deng et al., 2012). The correlation network at the genus level was drawn using Python.

## Results

### Identification and annotation of metabolites

A total of 2,424 metabolites were identified in the positive mode, and 1,542 metabolites in the negative mode. The metabolites in both modes were combined together, after which the KEGG, HMDB, and LIPID MAPS databases were used to annotate all the metabolites. The quantification and annotation information of the metabolites is summarized in Supplementary Table S1. After checking the list of all the metabolites, the downstream metabolites of the degradation of uric acid were not detected.

### Change of termite hindgut metabolome after captivity

**FIGURE 1:**
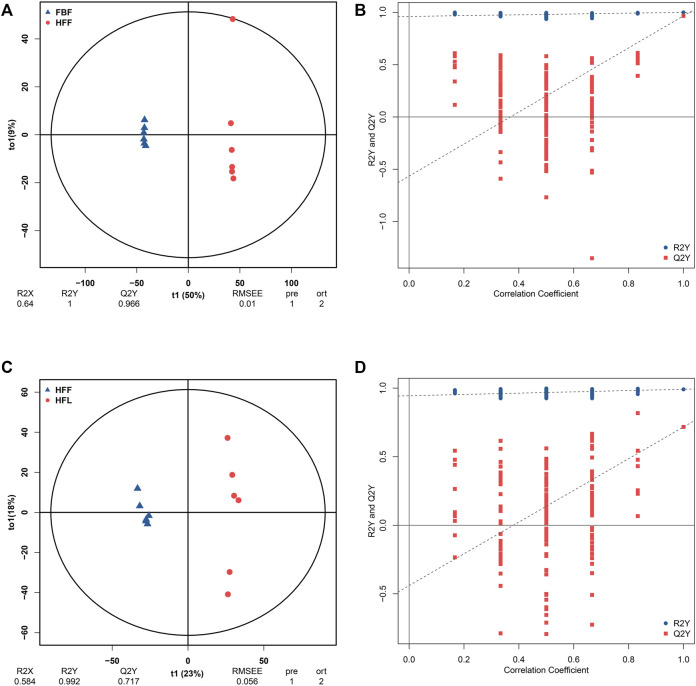
Results of the OPLS-DA analysis. **(A)** OPLS-DA score graph for FBF vs. HFF group. **(B)** OPLS-DA model permutation test graph for FBF vs. HFF group. **(C)** OPLS-DA score graph for HFF vs. HFL. **(D)** OPLS-DA model permutation test graph for HFF vs. HFL. In the OPLS-DA score graph, the x-axis (t1) represents the predicted component (the difference in components between groups), the y-axis (t2) represents the orthogonal component (the difference in components within group), and the percentage represents the proportion of the component in the total variance. In the OPLS-DA model permutation test graph, the x-axis represents the correlation coefficient between the permutation group and the original model group, the y-axis represents the value of R2Y or Q2Y, the blue and red dots represent the R2Y and Q2Y of the model after permutation respectively. Note: FBF = fat body of the freshly-collected workers; HFF = hindgut fluid of the freshly-collected workers; HFL = hindgut fluid of the laboratory-maintained workers.

FBF and HFF were separated clearly from each other on the OPLS-DA score plot (Figure 1A). Both the R2Y and Q2Y values exceeded 0.9 (R2Y = 1, Q2Y = 0.966), indicating the dependability of the OPLS-DA model. In addition, the result that R2Y value equaled to 1 represented the good accuracy of the OPLS-DA model. Therefore, the differentially abundant metabolites can be screened using VIP. According to the permutation test, the y-intercept of the Q2Y regression line was negative, which indicated that there was no overfitting in the OPLS-DA model (Figure 1B). To summarize, the original OPLS-DA model was quite robust, and was able to explain the metabolic difference between FBF and HFF. The fact that all the red dots were beneath the blue dots suggested that the training dataset and test dataset were relatively independent.

There was also a clear separation between HFF and HFL in the OPLS-DA plot (Figure 1C). The OPLS-DA model had good reliability and accuracy (R2Y = 0.992, Q2Y = 0.717). Therefore, the VIP can be used to screen the differentially abundant metabolites between HFF and HFL. No overfitting of the OPLS-DA model was observed according to the permutation test (Figure 1D). The HFF vs. HFL OPLS-DA also had considerable robustness. In addition, the training dataset was relatively independent of the test dataset, as all the red dots were below the blue dots.

**FIGURE 2:**
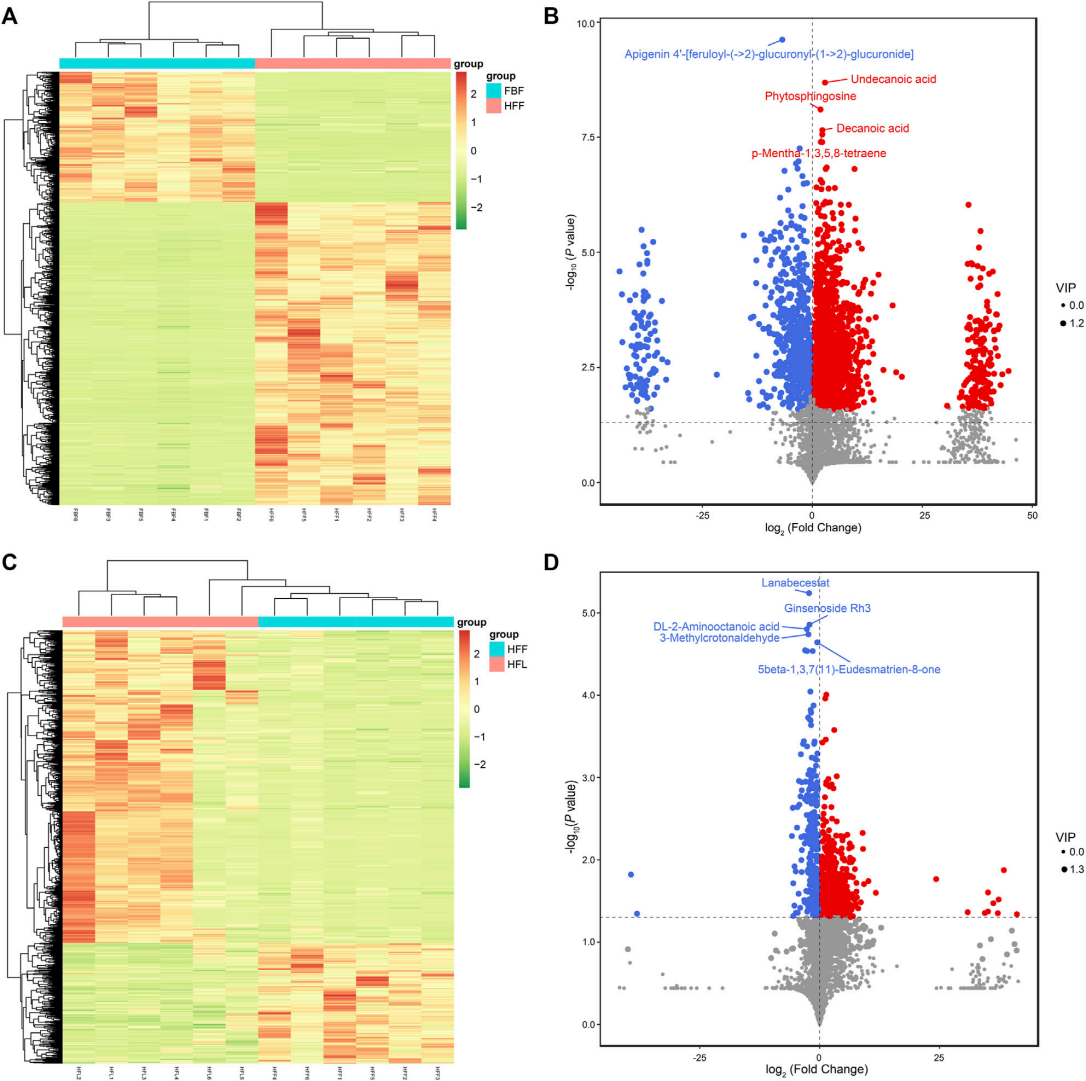
Clustering analysis and volcano plots of the differentially abundant metabolites. **(A)** Clustering analysis of all the differentially abundant metabolites in all the replicates for FBF vs. HFF group. **(B)** Volcano plot of all metabolites in FBF vs. HFF. **(C)** Clustering analysis of all the differentially abundant metabolites in all the replicates for HFF vs. HFL group. **(D)** Volcano plot of all metabolites in HFF vs. HFL. In the heatmap, each column represents a sample, and each row represents a metabolite. The metabolite abundance was z-score normalized. In the volcano plot, the red dot represents metabolite with increased abundance, the blue dot represents metabolite with reduced abundance, and the grey dot represents metabolite with unaltered abundance, the x-axis represents the fold change of each metabolite (log_2_ transformed), the y-axis represents the *P*-value of the t-test (-log_10_ transformed), and the size of a dot represents the VIP value of the OPLS-DA model. Note: FBF = fat body of the freshly-collected workers; HFF = hindgut fluid of the freshly-collected workers; HFL = hindgut fluid of the laboratory-maintained workers.

According to the cluster analysis of the differentially abundant metabolites, the six replicates from FBF were more likely to cluster together, while the six replicates from HFF formed their own cluster (Figure 2A). There were 1,443 metabolites with increased abundance, 621 with reduced abundance, and 1,902 with unaltered abundance in FBF, with HFF as a reference (Figure 2B). The differentially abundant metabolites between FBF and HFF are summarized in Supplementary Table S2. The concentrations of the amino acids, including L-valine, L-tyrosine, and L-arginine, were higher in HFF compared with that in FBF. D-fructose 1-phosphate and shikimic acid, the precursors for phenylalanine, tyrosine and tryptophan biosynthesis, were also more concentrated in HFF than in FBF. In addition, the higher concentrations of metabolites in HFF were also observed for the vitamins, cofactors, and their precursors, including vitamin K1, vitamin A, NADP, coenzyme B, (1R,6R)-6-hydroxy-2-succinylcyclohexa-2,4-diene-1-carboxylate, *γ*-tocotrienol, and 7,8-dihydrobiopterin. Some metabolites associated with fatty acid biosynthesis also had higher concentrations in HFF, including *α*-linolenic acid, oleic acid, decanoic acid, and stearic acid. In addition, the concentrations of two antibiotics (cephamycin C and geneticin) were higher in HFF compared with that in FBF. Finally, certain metabolites associated with the ovarian steroidogenesis and steroid biosynthesis were more concentrated in HFF than in FBF, including estrone glucuronide, 21-hydroxypregnenolone, pregnenolone, 15 (S)-HpETE, and testosterone.

The cluster analysis of the differentially abundant metabolites revealed that the six replicates of HFF were more likely to cluster together (Figure 2C). By comparing metabolites of HFF and HFL, 691 upregulated metabolites, 267 downregulated metabolites, and 3,008 unchanged metabolites were observed in HFF, with HFL as a reference (Figure 2D). The differentially abundant metabolites between HFF and HFL are summarized in Supplementary Table S3. The concentrations of metabolites, which were higher in HFF than in FBF, increased in HFL after captivity, including but not limited to vitamin A and metabolites in the steroid hormone biosynthesis and ovarian steroidogenesis [estrone glucuronide, 21-hydroxypregnenolone, and 15 (S)-HpETE]. However, for some other metabolites whose concentrations were higher in HFF than in FBF, their concentrations decreased after captivity. These metabolites included the following metabolites: L-tyrosine, cephamycin C, two metabolites in the amino sugar and nucleotide sugar metabolism [UDP-N-acetyl-*α*-D-glucosamine (UDP-GlcNAc) and guanosine diphosphate mannose], metabolites associated with the ubiquinone and other terpenoid-quinone biosynthesis [*γ*-tocotrienol and (1R,6R)-6-hydroxy-2-succinylcyclohexa-2,4-diene-1-carboxylate], and metabolites associated with the carbon metabolism (coenzyme B and gluconolactone). Meanwhile, the metabolites associated with the steroid hormone biosynthesis, including cortexolone, 7a-hydroxyandrost-4-ene-3,17-dione, dehydroepiandrosterone, and trans-dehydroandrosterone, increased in concentration in HFL compared with that in HFF. However, no significant difference was observed for the above four metabolites between FBF and HFF. An antibiotic, neamine, also became more concentrated in HFL compared with that in HFF.

**FIGURE 3:**
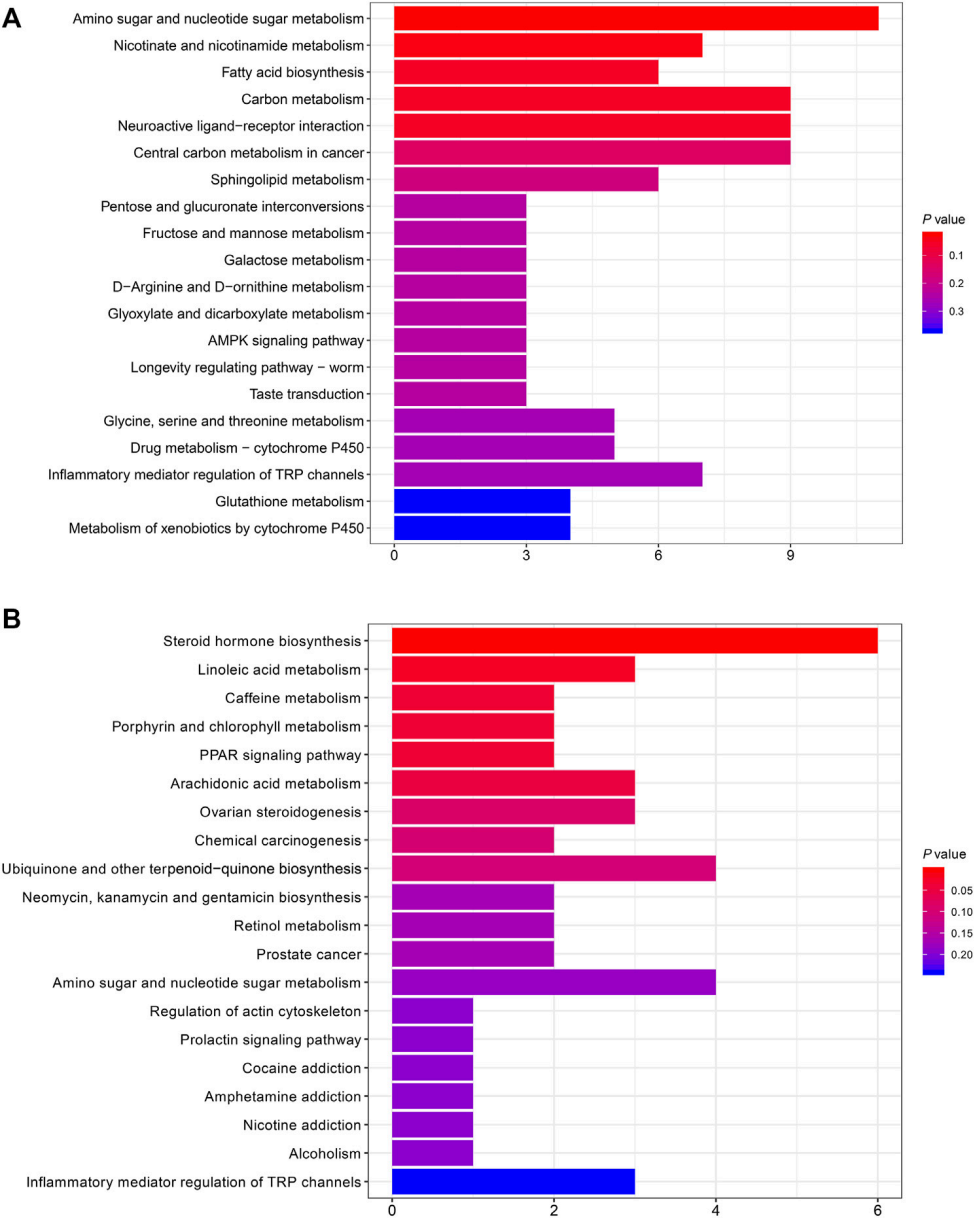
Bar plots that show the top 20 enriched KEGG pathways. **(A)** The top 20 enriched KEGG pathways in FBF vs. HFF. **(B)** The top 20 enriched KEGG pathways in HFF vs. HFL. The x-axis is the number of differentially abundant metabolites annotated to the pathway, and the y-axis is the pathway name. Note: FBF = fat body of the freshly-collected workers; HFF = hindgut fluid of the freshly-collected workers; HFL = hindgut fluid of the laboratory-maintained workers.

The KEGG pathway enrichment analysis was performed on the differentially abundant metabolites between FBF and HFF. According to the analysis, there were two enriched pathways, including amino sugar and nucleotide sugar metabolism (*P* = 0.025), and nicotinate and nicotinamide metabolism (*P* = 0.034) (Figure 3A). The differentially abundant metabolites between HFF and HFL were also subjected to KEGG pathway enrichment analysis. There were five pathways enriched according to the analysis, including steroid hormone biosynthesis (*P* = 0.002), linoleic acid metabolism (*P* = 0.024), caffeine metabolism (*P* = 0.037), porphyrin and chlorophyll metabolism (*P* = 0.037), and PPAR signaling pathway (*P* = 0.037) (Figure 3B).

### Sequencing and taxonomic classification of termite hindgut bacteria

A total of 116,869 raw CCS reads was generated through barcode sequences identification (Supplementary Table S4). The average number of the raw CCS reads in the F group (11,689 ± 414) was similar with that in the L group (11,685 ± 847). Then, the primer sequences and chimeric reads were removed. After length filtration, 99.2% ± 0.2% of effective CCS reads was obtained in F group, 98.7% ± 0.4% in L group. The average length of the clean CCS reads was 1,466 bp in F group, and 1,464 bp in L group.

**FIGURE 4:**
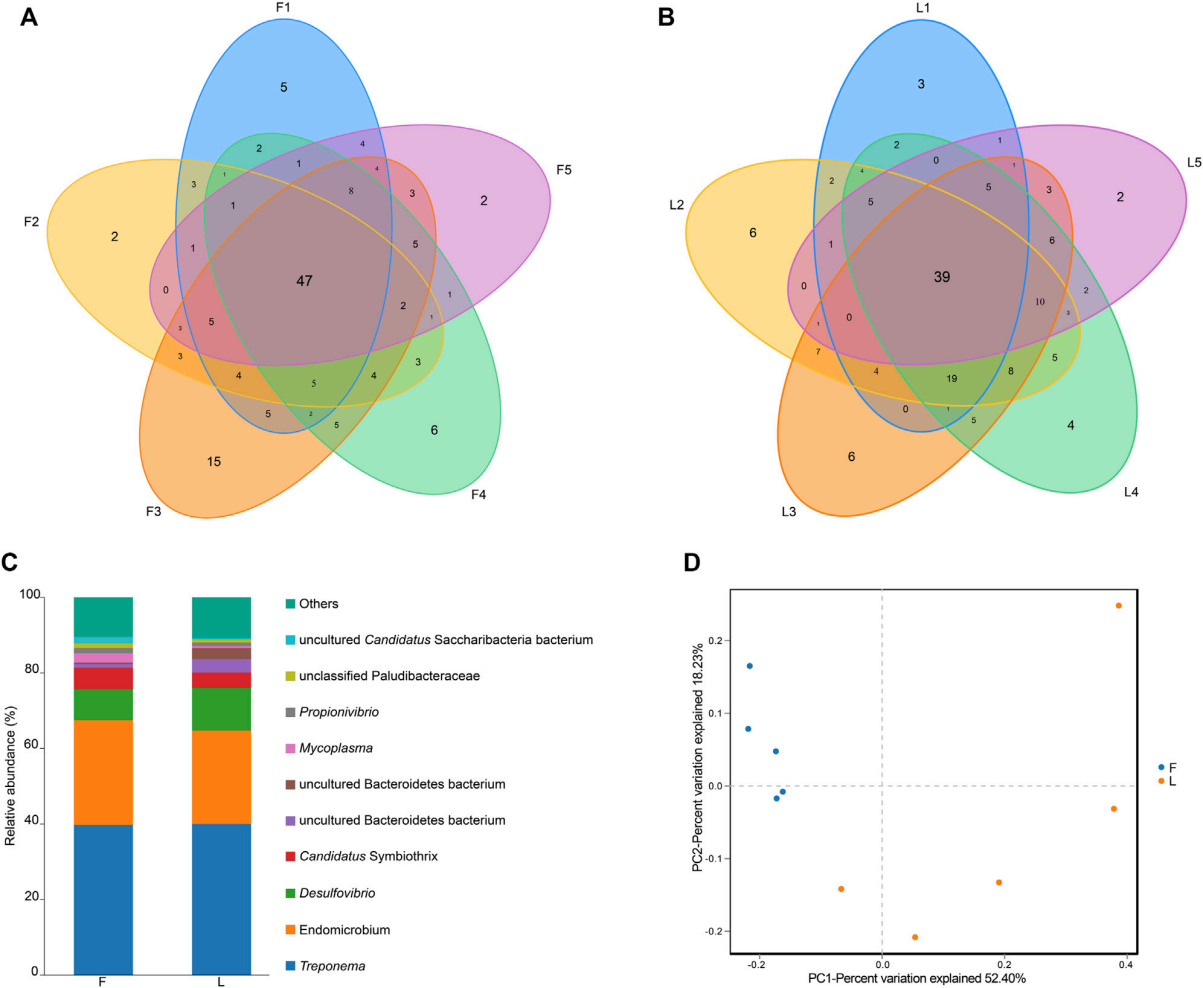
OTU analysis and taxonomic analysis. **(A)** Venn diagram presenting the number of OTUs in each sample in the F group. **(B)** Venn diagram indicating the number of OTUs in each sample in the L group. **(C)** Genus level relative richness in F and L group. x-axis represents the name of the group, and y-axis represents relative abundance of each genus. Each color block represents a genus. The area of a block represents relative abundance of a genus. **(D)** Visualization of the beta diversity analysis using principal coordinates analysis. Note: F = freshly-collected termites, L = laboratory-maintained termites.

A total number of 166 bacterial OTUs were identified by the 16S amplicon sequencing. The number of OTUs in each sample is shown in in Supplementary Figure S1. The highest was found in a F group (*n* = 120), while the lowest number of OTUs was found in a L group (*n* = 79). The F group shared 47 OTUs in common, while 39 OTUs were shared by the L group (Figures 4A, B).

Distribution of the genera for the F group and the L group is summarized in Figure 4C. For clear presentation of the data, only the top 10 genera were shown in the graph, and the rest were combined as others. *Treponema* was the most abundant genus in both the F group and the L group. The second and third largest genera in both groups were *Endomicrobium* and *Desulfovibrio*, respectively.

### Change of termite hindgut bacteria after captivity

The alpha diversity indices are summarized in Table 1. No significant differences between F and L group were observed for all the five alpha diversity indices (*P* > 0.05). The beta diversity of between groups was assessed using PCoA analysis. A clear separation between F and L group was observed. According to the PCoA analysis, PC1, PC2, and PC3 accounted for the 52.40%, 18.23%, and 11.46% of the variation, respectively. The samples from F group clustered more closely than those from L group (Figure 4D).

**TABLE 1:** Alpha diversity indices of the hindgut bacteria from the freshly-collected termites (F) and those from the laboratory-maintained termites (L).

Alpha diversity indices	F	L
Chao1	114.59 ± 7.32	126.17 ± 15.74
ACE	121.80 ± 6.96	124.55 ± 6.89
Shannon	4.13 ± 0.16	3.77 ± 0.18
Simpson	0.89 ± 0.01	0.85 ± 0.02
PD whole tree	10.09 ± 0.44	10.40 ± 0.61

**FIGURE 5:**
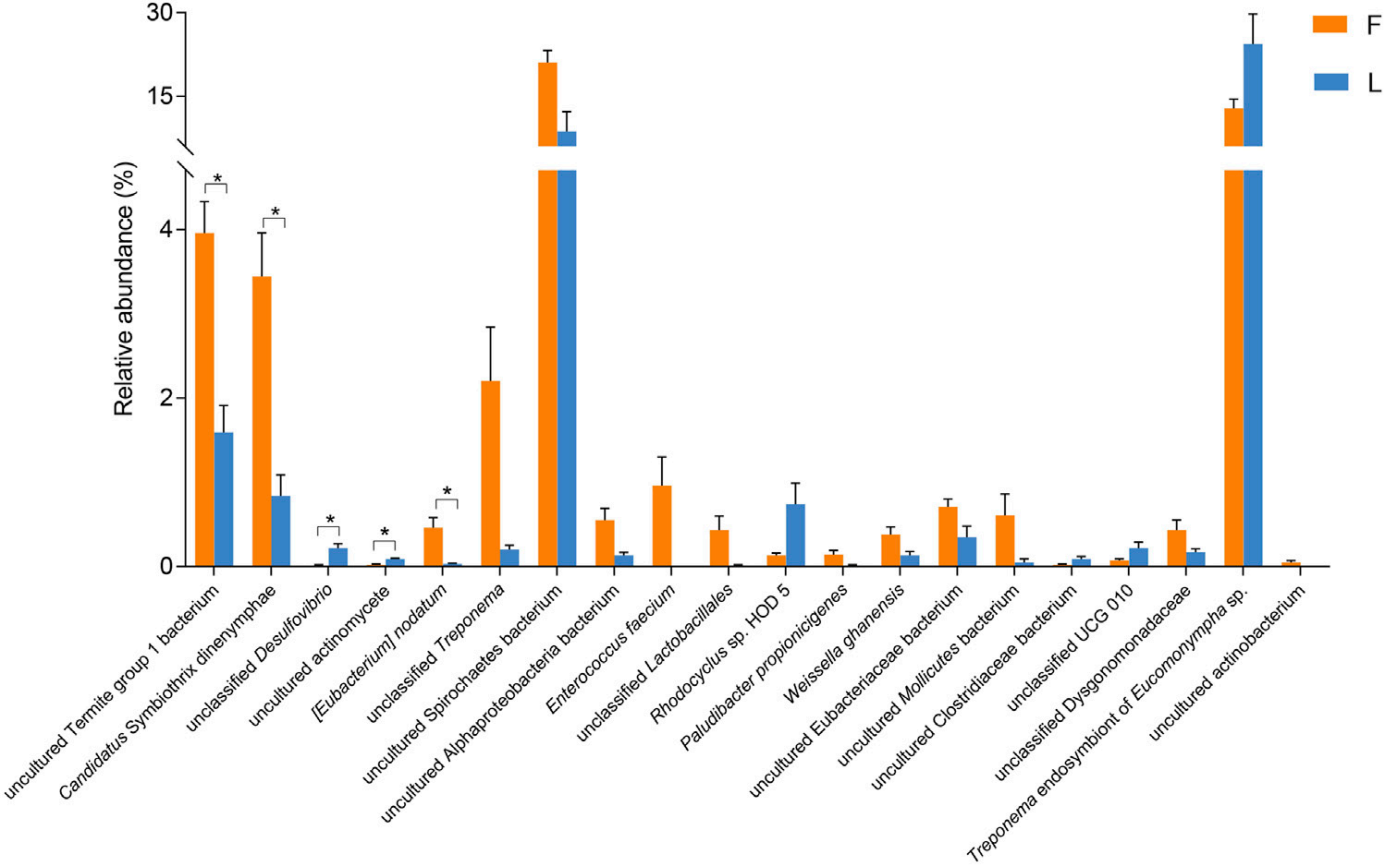
Comparison of the hindgut bacteria abundance between F and L group at the species level. The differential analysis was performed using Metastats. Only the top 20 species with the lowest *Q* values are shown. Note: F = freshly-collected termites, L = laboratory-maintained termites.

**FIGURE 6:**
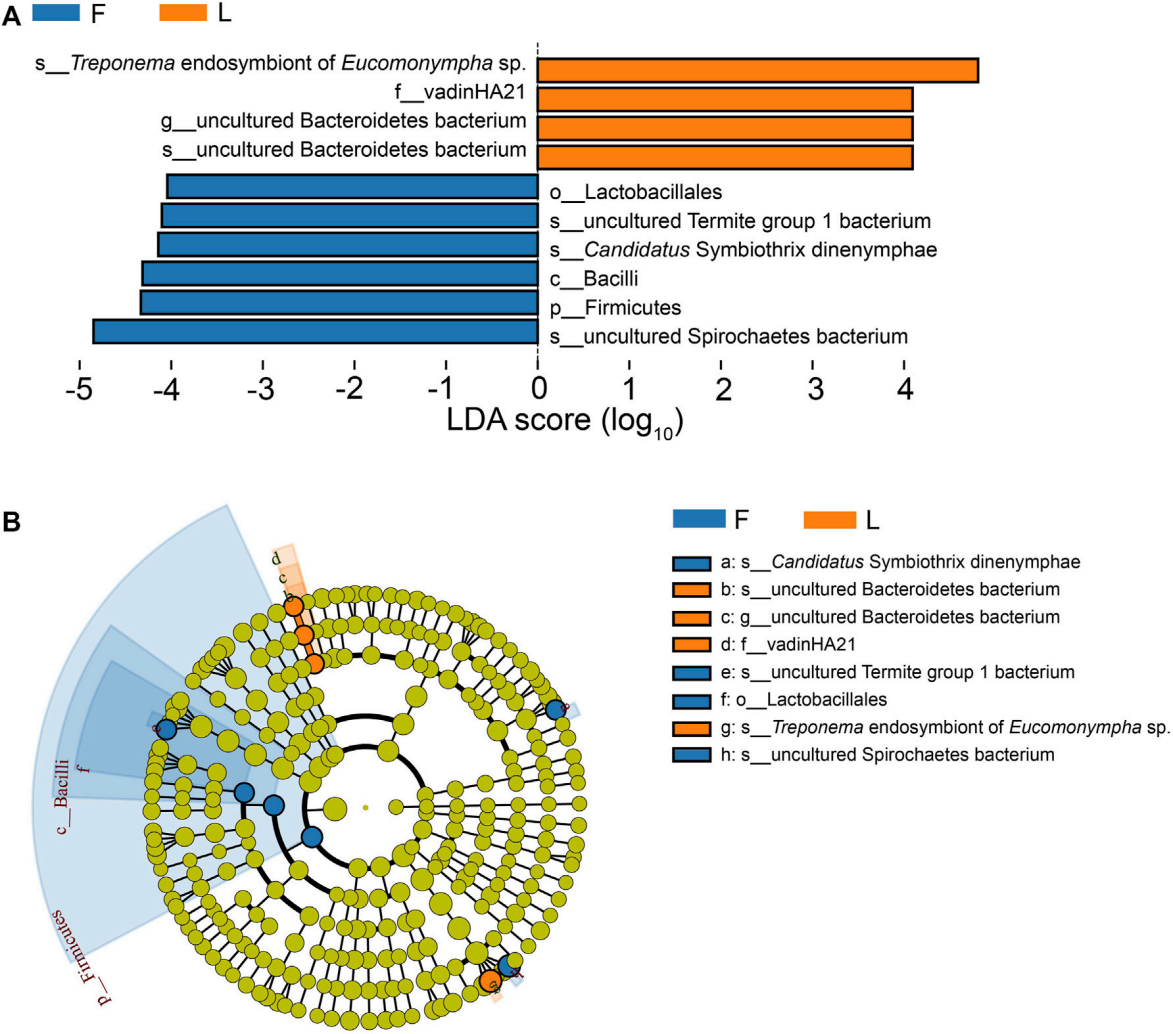
Differentially abundant taxa between the F group and the L group identified by LEfSe analysis. **(A)** Histogram of LDA score. The taxa whose LDA scores are higher than the set value are shown in the graph (the default is 4.0). The impact of different taxa (i.e., LDA Score) is shown by the length of the bar, and different colors reflect taxa in different groups. **(B)** Cladogram based on LEfSe analysis. In the cladogram, the taxonomic categories from phylum to species are represented by circles from inner to outer. Relative abundance is indicated by the size of the node. Green color represents the taxa which have no significant difference. Note: F = freshly-collected termites, L = laboratory-maintained termites.

**FIGURE 7:**
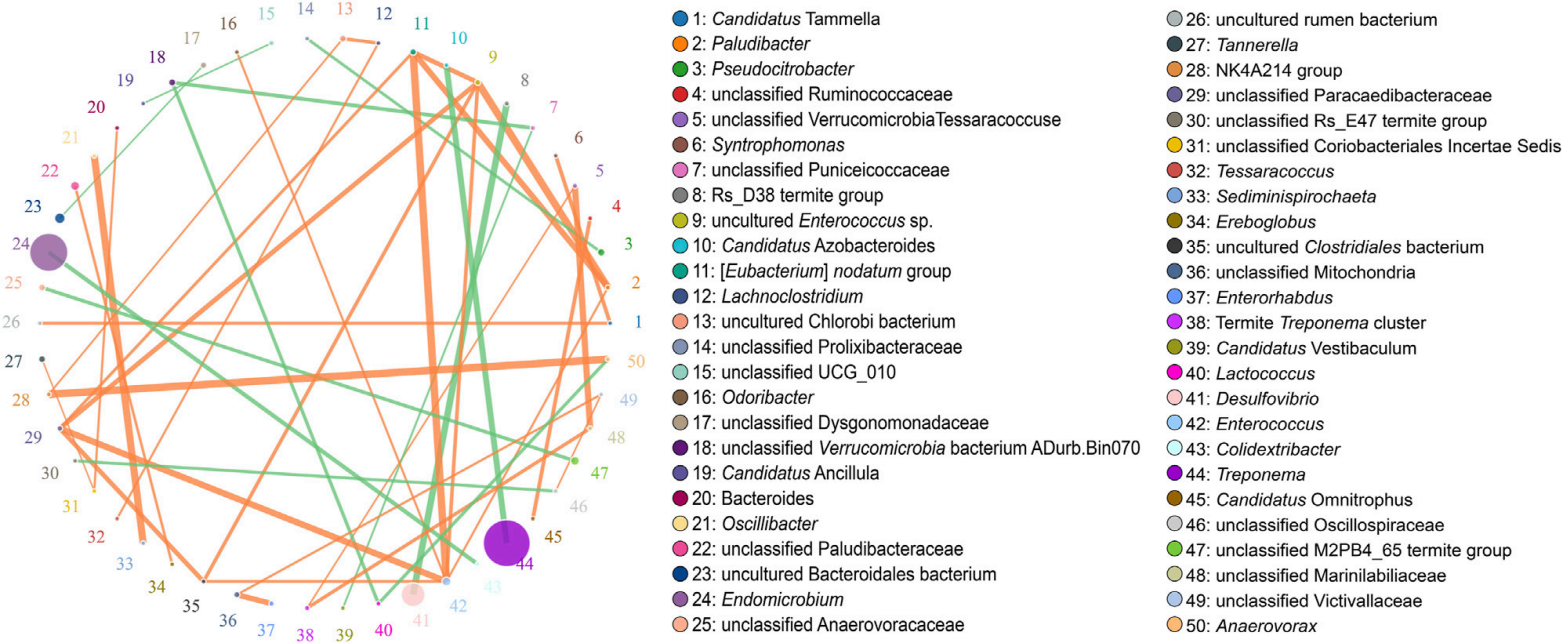
Correlation network at the genus level. The size of the circle denotes the abundance, and the size of the circle represents the genera. The association between the two genera is represented by the line. The correlation’s strength is represented by the line’s thickness. Positive correlation is represented by orange, while negative correlation is represented by green.

According to the Metastats analysis, the abundance of five species was significantly different between F group and L group: uncultured Termite Group 1 (TG1) bacterium (*Q* = 0.03), *Candidatus* Symbiothrix dinenymphae (*Q* = 0.03), unclassified *Desulfovibrio* (*Q* = 0.03), uncultured actinomycete (*Q* = 0.03), and *Eubacterium nodatum* (*Q* = 0.04) (Figure 5). There were six discriminative features in F group, and four in L group (Figure 6A). The cladogram based on LEfSe analysis is shown in Figure 6B. According to the LEfSe analysis, the species which served a significant role in the L group, including *Treponema* endosymbiont of *Euconympha* sp. and uncultured Bacteroidetes bacterium. In the F group, three species were recognized as significant biomarkers, including uncultured TG1 bacterium, *Ca.* S. dinenymphae, and uncultured Spirochaetes bacterium. According to correlation analysis, the most abundant genus *Treponema* was negatively correlated with *Candidatus* Azobacteroides. The second most abundant genus *Endomicrobium* was negatively correlated with *Colidextribacter*. None of the two genera showed positive correlation with other genera (Figure 7).

## Discussion

The hindgut of termite provides a highly structured microenvironment for the microbiota, rendering the microbiota difficult to culture (Brune and Friedrich, 2000; Li et al., 2012). Therefore, the function of the microbiota is primarily inferred from the genome, transcriptome or the amplicon sequencing study (Scharf and Peterson, 2021). The comparison of the metabolites between FBF and HFF provides another piece of information about the function of the hindgut microbiota. The concentrations of certain amino acids and their precursors were higher in HFF compared with those in FBF, including L-tyrosine, L-arginine, and L-valine. Besides amino acids, higher concentrations of fatty acids (linolenic acid, oleic acid, decanoic acid, and stearic acid), vitamins (vitamin A and vitamin K1), and cofactors (NADP and coenzyme B) were also observed in HFF compared with those of FBF. These findings suggest that the hindgut microbiota provides important nutritional factors to the host, including amino acid, fatty acid, and cofactors. However, there is another possibility that these nutritional factors are produced by the host through host-microbiota interactions in the hindgut. The metabolome study also provides direct evidence for the synthesis of antibiotics by the gut bacteria, as neamine and geneticin were detected. Our findings imply the importance of the hindgut microbiota, which was also observed in other insects. For example, in honey bee, the bacteria, which inhibit mostly in the hindguts, digest the pollen ingested by the hosts, and produce the organic acids and aromatic compound degradation intermediates (Kešnerová et al., 2017).

The comparison of metabolites between HFF and HFL reveals that captivity influences the metabolism in the hindguts. UDP-GlcNAc is the synthesis unit for chitin, which is the major component for cell wall. The concentration of UDP-GlcNAc was lower in HFL compared with that in HFF. It is suggested that captivity influence the synthesis of cell wall of the hindgut bacteria. Two metabolites of the carbon metabolism pathway (coenzyme B and gluconolactone) decreased in the hindguts after captivity of termites. Captivity can also influence the biosynthesis of tyrosine, and biosynthesis of ubiquinone and other terpenoid-quinone. The concentration of *γ*-tocotrienol, which is the immediate precursor for *α*-tocotrienol (vitamin E), was lower in HFL than that in HFF. The concentration of (1R,6R)-6-hydroxy-2-succinylcyclohexa-2,4-diene-1-carboxylate, which is the precursor for synthesis of vitamin K1 (phylloquinone) and vitamin K2 (menaquinone), also decreased after captivity. Therefore, the gut microbiota provides less nutritional factors to the host, leading to undernourishment of termites in captivity. The gut fluid of the termites in the field is brown, while that of the termites in captivity is white, indicating that termites in the filed may take in other food besides wood, such as soil (Janzow and Judd, 2015; Mullins et al., 2021). However, the termites in the captivity feed on wood only, so they may be in short of certain micronutrients from the soil, which influences the activity of certain enzyme.

Our study indicates that the hindgut microbiota may produce hormone to regulate the caste differentiation. After conducting KEGG enrichment analysis of the differentially abundant metabolites between HFF and HFL, we found several important metabolism pathways enriched, including steroid hormone biosynthesis. Through the screening of the differentially abundant metabolites between FBF and HFF, it was found that some metabolites associated with steroid hormone biosynthesis and ovarian steroidogenesis were more concentrated in HFF than in FBF, including estrone glucuronide, 21-hydroxypregnenolone, and 15 (S)-HpETE. The concentrations of these metabolites further increased in HFL after captivity. The change of these metabolites mentioned above reflected the formation of secondary reproductives at the end of containment. Although only the hindgut fluid from the workers were collected for metabolome study, the metabolites related to steroid hormone biosynthesis and ovarian steroidogenesis were still detected. These metabolites may be transmitted from the secondary reproductives to workers through trophallaxis. These metabolites were enriched in the hindgut fluid when compared with the fat body, indicating that these metabolites are produced by the hindgut microbiota. Our research provides another evidence to the role of hindgut microbiota on caste differentiation (Scharf and Peterson, 2021).

Another obvious change when termites are captivated in the laboratory is the accumulation of uric acid. Uric acid plays a variety of roles in insect. It serves as an antioxidant to enhance the longevity of termites (Tasaki et al., 2017). However, as the concentration of uric acid gradually increases in the fat body after containment of termites, the accumulation of uric acid can even lead to the death of laboratory-maintained termites according to our observation. Cockroaches and termite *Mastotermes darwiniensis* possess mycetocytes in the fat body (Bandi and Sacchi, 2000). *Blattabacterium*, a rod-shaped uricolytic bacterium, is present in mycetocytes (Bandi and Sacchi, 2000). However, *H. sjostedi* lacks mycetocytes, and therefore there are no uricolytic bacteria in the fat body. According to Potrikus and Breznak (Potrikus and Breznak, 1980b), uricase activity was not found in termites. *Reticulitermes flavipes* utilizes the hindgut bacteria to degrade uric acid (Potrikus and Breznak, 1981). Therefore, it was speculated that a way *H. sjostedi* to degrade uric acid is through the hindgut bacteria. The bacteria from the genera *Pseudomonas*, *Enterobacter*, and *Lactococcus* were isolated from the gut of the snail *Pomacea canaliculata* and their role of uric acid degradation has been proved (Koch et al., 2014). Bacteria, such as N *Streptococcus* sp., *Bacteroides termitidis*, and *Citrobacter* sp., have the ability to degrade uric acid in *R. flavipes* (Potrikus and Breznak, 1980a). Actinobacteria isolated from the gut of *Reticulitermes* sp. are capable of degrading uric acid *in vitro* (Arango et al., 2017). However, according to the 16S amplicon study, these bacteria were not detected. According to the function prediction of the gut bacteria, the genomes of the all the detected bacteria do not encode the uricase. In addition, the metabolites at the downstream of uric acid degradation pathway, such as 5-hydroxyisourate, allantoin, and allantoate, were not detected in both HFF and HFL according to the metabolome data. *H. sjostedi* may use other mechanisms to utilize uric acid (Konishi et al., 2023).

The abundance of uncultured TG1 bacterium was higher in F group than that in L group. According to the LEfSe analysis, the uncultured TG1 bacterium was recognized as significant biomarker in the freshly-collected termites. Many cellulolytic gut protists harbor TG1 bacteria as intracellular symbionts (Ohkuma et al., 2007). The TG1 bacteria serve as crucial symbionts for the cellulolytic protists, providing stable supplies of the nitrogenous compounds to their host protists. Another different bacterium was uncultured *Ca.* S. dinenymphae, which was also identified by the LEfSe analysis as a discriminative feature in the freshly-collected termites. *Ca.* S. dinenymphae is an ectosymbiont of protists from the genus *Dinenympha* (Hongoh et al., 2007). The genome of *Ca.* S. dinenymphae reveals that it encodes the enzyme for lignocellulose digestion and amino acids biosynthesis (Yuki et al., 2015). According to the metabolome data, the concentrations of several nutritional factors, such as (1R,6R)-6-hydroxy-2-succinylcyclohexa-2,4-diene-1-carboxylate, *γ*-tocotrienol, and tyrosine, were higher in the guts of freshly-collected termites compared to the laboratory-maintained termites. The combination of metabolome study and 16S amplicon study supports the view that those bacteria or their host protists could provide nutritional factors to the host.

The third biomarker which was identified as playing an important role in the freshly-collected termites was an uncultured Spirochaetes bacterium. Spirochaetes are one of the most abundant bacteria in termites’ guts. They may live in the free state in the hindgut fluid, or live as the ectosymbionts or endosymbionts of protists. Most of the Spirochaetes identified from termite guts are from the genus *Treponema* (Lilburn et al., 1999). Spirochetes from hindguts of *Zootermopsis angusticollis* synthesize acetate from H_2_ plus CO_2_ (Leadbetter et al., 1999). They also show nitrogenase activity and participate in nitrogen fixation (Lilburn et al., 2001). The large protist *Eucomonympha* in the *H. sjostedi* harbors an endosymbiont from the genus *Treponema*, which also performs acetogenesis and nitrogen fixation (Ohkuma et al., 2015). *Treponema* endosymbiont of *Eucomonympha* sp. served an important function in the laboratory-maintained termites according to the LEfSe analysis. After containment of termites in the laboratory, nitrogen may be a limiting factor for termites. Termites may increase the activity of nitrogen fixation to maintain the nitrogen balance. However, although *C. formosanus* harbors the nitrogen fixation bacteria, in the incipient colonies of *C. formosanus* nitrogen fixation does not occur (Mullins and Su, 2018). The reason for the change of the Spirochaetes after captivity of termites has to be further confirmed.

## Conclusion

Due to the complexity of the microenvironment in the hindgut of termites, the microbiota is difficult to culture (Hongoh, 2010). Thus, the omics methods have been widely used to study the function of the hindgut microbiota (Warnecke, et al., 2007; Hongoh et al., 2008; Ohkuma et al., 2015; Liu, et al., 2019; Calusinska et al., 2020; Nishimura et al., 2020). In this study, the combination of metabolome study and 16S amplicon sequencing was used to investigate the function of the hindgut microbiota. Our research provides evidence that the hindgut microbiota can provide crucial nutritional factors for the host, such as amino acid, vitamin, fatty acids, and cofactors. In other words, our study has shown that the hindgut microbiota is an indispensable and vital part of termites. In addition, termites maintain a core microbiota to preserve the function of the hindgut microbiota. However, captivity influences the hindgut microbiota, which reflets the selective pressure imposed by the captivity of termite in the laboratory. Therefore, our results also point out the importance of using the freshly-collected termites to reflect the hindgut microbiota composition of the field colonies (Husseneder et al., 2009).

## Data Availability

The original contributions presented in the study are publicly available. This data can be found here: https://www.ncbi.nlm.nih.gov/search/all/?term=PRJNA952986.

## Supplementary material

SUPPLEMENTARY TABLE S1 The quantification and annotation information of the metabolites in FBF, HFF and HFL. Note: FBF, fat body of the freshly-collected termites; HFF, hindgut fluid of the freshly-collected termites; HFL, hindgut fluid of the laboratory-reared termites.

SUPPLEMENTARY TABLE S2 The differentially abundant metabolites between FBF and HFF. Note: FBF, fat body of the freshly-collected termites; HFF, hindgut fluid of the freshly-collected termites.

SUPPLEMENTARY TABLE S3 The differentially abundant metabolites between HFF and HFL. Note: HFF, hindgut fluid of the freshly-collected termites; HFL, hindgut fluid of the laboratory-reared termites.

SUPPLEMENTARY TABLE S4 Sequencing quality of the 16S rRNA gene sequencing study. F1-F5 were the five replicates of the freshly-collected termites (F), while L1-L5 were the five replicates of the laboratory-maintained termites (L).

SUPPLEMENTARY FIGURE S1 The number of OTUs in each sample of the hindgut bacteria community study. F1-F5 were the five replicates of the freshly-collected termites (F), while L1-L5 were the five replicates of the laboratory-maintained termites (L).
