# Supplementary figures and images for: Change of termite hindgut metabolome and bacteria after captivity indicates the hindgut microbiota provides nutritional factors to the host
Source: Front Bioeng Biotechnol. 2024 Jan 15;11:1228918. doi: 10.3389/fbioe.2023.1228918 (PMC10823432; doi:10.3389/fbioe.2023.1228918)

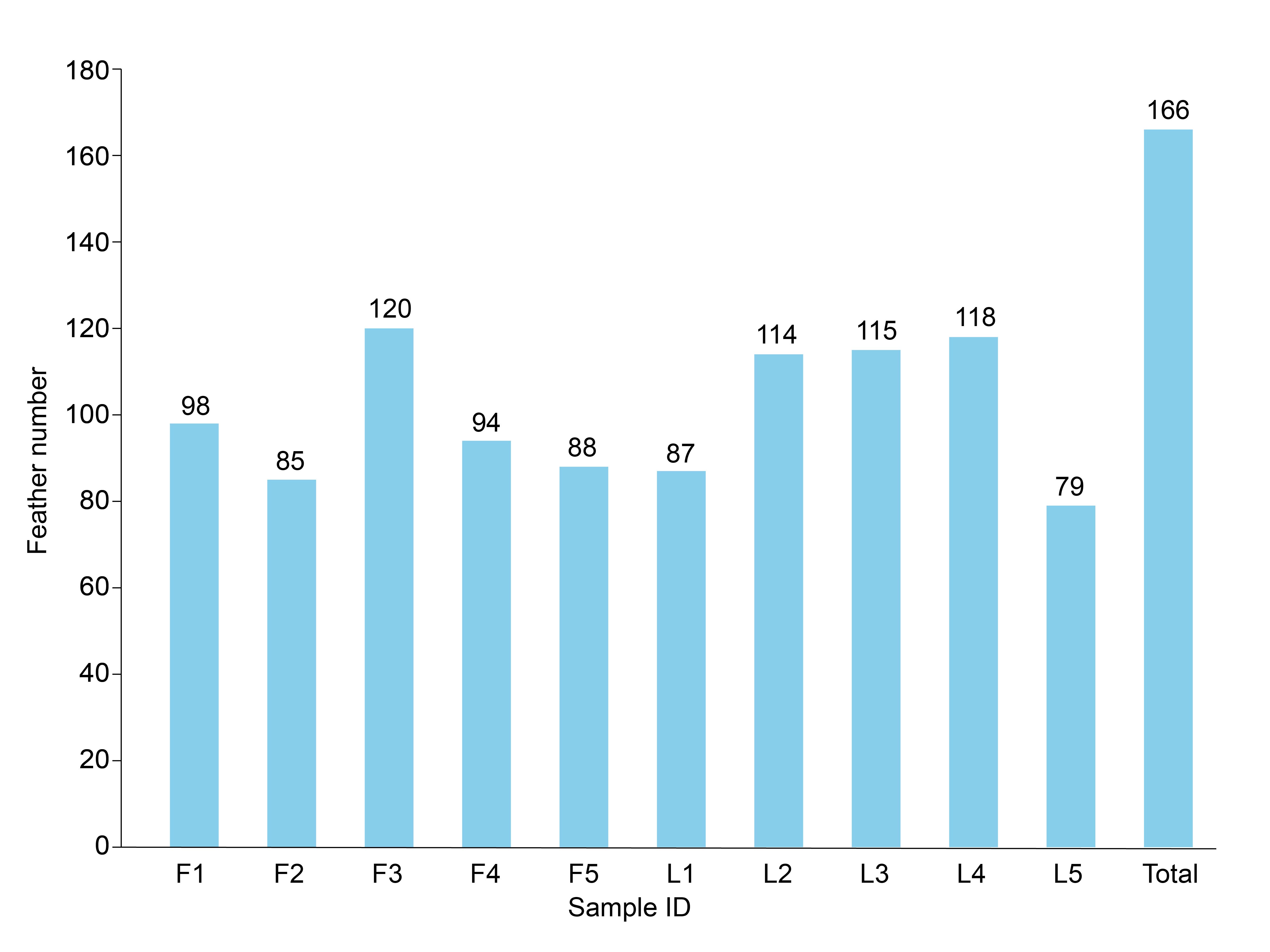

Supplement: Supplementary file 3 [file Image1.JPEG]
